# Protocol for the pilot randomized trial of the *CA*rdiovascular *R*isk ass*E*ssment for *R*heumatoid *A*rthritis (CARE RA) intervention: a peer coach behavioral intervention

**DOI:** 10.1186/s40814-022-01041-z

**Published:** 2022-04-15

**Authors:** Joan Weiner, Geyanne Lui, Mackenzie Brown, Yuliana Domínguez Páez, Shelley Fritz, Tien Sydnor-Campbell, Aberdeen Allen, Assem Jabri, Shilpa Venkatachalam, Kelly Gavigan, William Benjamin Nowell, Jeffrey R. Curtis, Liana Fraenkel, Monika Safford, Iris Navarro-Millán

**Affiliations:** 1 Indiana University, Bloomington, IN USA; 2 Peer Coach CARE RA, New York, USA; 3 New York Institute of Technology College of Osteopathic Medicine, Old Westbury, NY USA; 4 Division of General Internal Medicine, Weill Cornell Medicine, New York, NY USA; 5 BodyMind Consulting, LLC, Philadelphia, USA; 6 Colgate Palmolive, New York, USA; 7 Global Healthy Living Foundation, Upper Nyack, NY USA; 8 Universtiy of Alabama at Birmingham, Birmingham, AL USA; 9 Yale University, New Haven, CT USA; 10 Berkshire Medical Center, Berkshire, Pittsfield, MA USA; 11 Division of Rheumatology, Hospital for Special Surgery, New York, NY USA

**Keywords:** Peer coaches, Implementation science, Cardiovascular disease, Rheumatoid arthritis

## Abstract

**Background:**

Cardiovascular disease (CVD) is the most common cause of death among people with rheumatoid arthritis (RA), with an estimated increased risk of 50–60% compared to the general population. Lipid-lowering strategies have been shown to lower CVD risk significantly in people with RA and hyperlipidemia. Thus, CVD risk assessment has an important role to play in reducing CVD among people with RA. Yet currently only 37 to 45% of this population are receiving primary lipids screening. This paper describes the CArdiovascular Risk assEssment for RA (CARE RA) intervention, which is designed to address this issue. CARE RA is a peer coach intervention, that is, an intervention in which a person with RA coaches another person with RA, which is designed to educate people with RA about the relation between RA and CVD risk and to help them obtain evidence-based CVD risk assessment and treatment.

**Methods:**

This is an open-label pilot study that will test if the participants assigned to complete the CARE RA curriculum with a peer coach will receive a cardiovascular risk assessment more frequently compared to those that complete the CARE RA curriculum by themselves. The CARE RA intervention is guided by Social Cognitive Theory. Participants in the peer coach intervention arm will receive the assistance of a peer coach who will call the participants once a week for 5 weeks to go over the CARE RA curriculum and train them on how to obtain CVD risk assessment. The control arm will complete the CARE RA curriculum without any assistance. Participants will be randomized 1:1 either to the control arm or to the peer coach intervention arm. The primary outcome is a participant’s having a CVD risk assessment or initiating a statin, if indicated. Secondary outcomes include patient activation and RA medication adherence. The RE-AIM implementation framework guides the implementation and evaluation of the intervention.

**Discussion:**

This pilot study will test the feasibility of the peer coach intervention in anticipation of a larger trial. CARE RA pioneers the use of peer coaches to facilitate the implementation of evidence-based treatment guidelines among people with RA.

**Trial registration:**

ClinicalTrials.gov NCT04488497. Registered on July 28, 2020.

**Supplementary Information:**

The online version contains supplementary material available at 10.1186/s40814-022-01041-z.

## Contributions to the literature

- The Cardiovascular Risk assEssment for Rheumatoid Arthritis (CARE RA) will promote the 2018 ACC/AHA cholesterol treatment guidelines for people with rheumatoid arthritis (RA). These guidelines recommend a 10-year risk assessment for cardiovascular disease (CVD) as well as a list of criteria for initiating treatment with a statin.
- CARE RA will be the first investigation using peer coaches to help people with RA understand the increased CVD risk associated with RA.
- CARE RA will provide a framework for additional investigational strategies to inform people with RA about health risks and enable them to take an active role in their health care.

## Background

Cardiovascular disease (CVD) is the most common cause of death among people with rheumatoid arthritis (RA). The increased risk of death from myocardial infarction and stroke in people with RA compared with the general population has been estimated at 50–60%, with the standardized mortality ratio attributable to CVD ranging from 1.13 to 5.15 [1–6]. Aggressive treatment of RA has been shown to decrease CVD morbidity and mortality [7–11]. However, many people with RA do not receive RA treatment that is sufficiently aggressive to attenuate the excess CVD risk associated with RA [12]. Lipid-lowering strategies have also been shown to lower CVD risk significantly in people with RA and hyperlipidemia [7, 13]. The American College of Cardiology/American Heart Association (ACC/AHA) recommend cholesterol testing to assess CVD risk, and initiation of a statin if risk is above a recommended threshold for individuals without diabetes or established CVD (e.g., prior stroke or myocardial infarction) [8].

Despite the high risk for CVD among people with RA and evidence-based guidelines for primary CVD prevention, CVD risk assessment in this population remains suboptimal, with only between 37 and 45% of people with RA receiving lipids panels [9, 10]. Moreover, while many published interventions aimed at improving screening and initiating lipid-lowering treatment have been conducted among patients with established CVD or diabetes mellitus (DM), few CVD interventions have been focused on people with RA [11, 14–19]. One approach for increasing CVD risk assessment among people with RA would be to target physicians, because reports suggest there is under-recognition of CVD risk in patients with RA. However, physician-targeted interventions typically have modest effectiveness [11, 20]. Interventions targeted at physicians and incorporated into the electronic health record (EHR) have resulted in physicians reporting fatigue and dissatisfaction with the implementation of CVD risk reduction guidelines [11, 21]. In contrast, there is promising evidence for the efficacy of interventions aimed at stakeholders. For example, in a recent study employing professionally trained actors who were trained to portray characters experiencing depression, those who asked for a treatment for depression were nearly eight times as likely to receive a prescription for an antidepressant medication as those who made no request [22]. This is consistent with many studies demonstrating that prompting patients to ask their providers specific questions leads to changes in care [22–27]. Yet people with RA are typically unaware that excess CVD risk is a component of RA-related inflammation [28]. All this suggests that it may be useful to explore interventions that are aimed at people with RA. However, interventions that must be completed by patients on their own often lead to low engagement and completion rates, raising concerns about long-term sustainability. This low level of engagement has been reported to result from the requirement for high motivation on the part of the patient to complete these interventions [29, 30].

The use of peer coaches is a promising strategy for training people with RA to request appropriate testing and treatment. Peer coaches are lay individuals who have the targeted condition themselves and receive training on the issues of interest. Peer coach interventions have been shown to be successful for medication adherence among people with human immunodeficiency virus (HIV), asthma, and diabetes, and for cancer screening [31–38]. Other studies have shown counseling by trained or untrained peers to improve medication initiation, adherence, and outcomes in people experiencing asthma, coronary heart disease, and HIV [35, 36, 39, 40].

We designed a randomized control trial to pilot test the effectiveness of a peer coaching intervention, called the Cardiovascular Risk assEssment for Rheumatoid Arthritis (CARE RA), to overcome barriers to screening and treatment for CVD risk reduction among people with RA. This study will test the primary hypothesis that the proportion of people who have a CVD risk assessment, defined as having a discussion with their doctor about their cholesterol test results or having a cholesterol test if they did not have one at enrollment, or initiate a statin, if indicated, in the peer coach intervention group (CARE RA curriculum + activation by having guidance from a peer coach), will be higher than in the control group. The control group will be provided with the CARE RA curriculum materials but will not have peer coach guidance. Secondary objectives are to determine if patient activation, self-efficacy, and RA medication adherence are higher in the peer coach arm than in the control arm. Implementation objectives will include reach, feasibility, implementation, adoption, fidelity, and maintenance of CARE RA. The evaluation of the peer coach intervention is guided by the Reach, Effectiveness, Adoption, Implementation, Maintenance (RE-AIM) Framework [41].

## Methods

### Theoretical framework and implementation framework

The CARE RA peer coach intervention is guided by Social Cognitive Theory (SCT) [42]. This theory posits three mechanisms of human agency: direct personal agency (self-efficacy), proxy agency (reliance on others, such as parents or partners, acting at one’s behest to secure desired outcomes), and collective agency (coordinated interdependent efforts). Table 1 lists barriers to effective participation of people with RA in their care and demonstrates how the intervention maps to SCT. It also maps each CARE RA program session’s activities and targeted barriers to each construct of this theory. Additional file 1 has detailed information about the measures that will be collected in the study and the tenets of the RE-AIM implementation and evaluation framework that we will use in this study.

**Table 1:** CARE RA peer coach intervention mapping to Social Cognitive Theory

Theoretical construct	Targeted barrier	Intervention activity	Corresponding session
**Self-Efficacy**	Feeling isolated	Supportive Coaching	Session 1, 2, 3 *Learn More* modules
	Lack of understanding of CVD risk	Education (PALS)	
	Lack of understanding of the effects of RA systemically	Education	
	Lack of understanding of the value of having a PCP	Education	
**Outcome Expectation**	Unrealistic expectations about the goals for RA disease control	Education	Sessions 2, 3, 4 *Learn More modules*
	Unrealistic expectations about their CVD risk	Education	
	Unrealistic expectations that because they ask for a cholesterol test, that the doctor will order it	Action planning	
	Fear of need to take another medication	MoI	
**Socio-Cultural Factors**	No resources to learn from about CVD	Supportive coaching	Session 2, 3, 4, 5 *Learn More* modules
	No guidance of how to engage in healthy behaviors (e.g., healthy diet, regular exercise, take medications)	Coaching	
	Disruptive social support (e.g., family preference for unhealthy diet, preference for junk food than healthy food)	Coaching	

*MoI* motivational interviewing, *CVD* cardiovascular disease, *PCP* primary care provider, *PALS* Patient Activated Learning System

### Study population

The study will include people with RA between the ages of 40 and 75 years (the age range of the current ACC/AHA guidelines for primary CVD risk assessment) [43] who have no history of CVD, no history of diabetes, and are not currently receiving a statin or do not have a recollection of discussing their CVD risk or cholesterol test results with any of their doctors. In addition to these requirements, all participants must satisfy the following inclusion criteria: provide a date of their next appointment with their rheumatologist that is within approximately the next 4–6 weeks, be willing to work with a peer coach, speak English, have a phone, have access to the Internet, have a personal email address, and reside in the United States. Prospective participants with dementia or severe cognitive decline are de facto excluded due to the relative complexity of the enrollment procedure. Participants will not be excluded on the grounds of current or prior COVID infection.

A small group (*n* = 8) of people with RA in the same age group as participants will be trained as peer coaches and be co-investigators in the study. They should have had a cardiovascular risk assessment done including having a cholesterol test or they should complete this process while getting training. Four of the proposed peer coaches have already been chosen, trained, and involved in the design of this study.

### Study design

#### Trial design

**Fig. 1:**
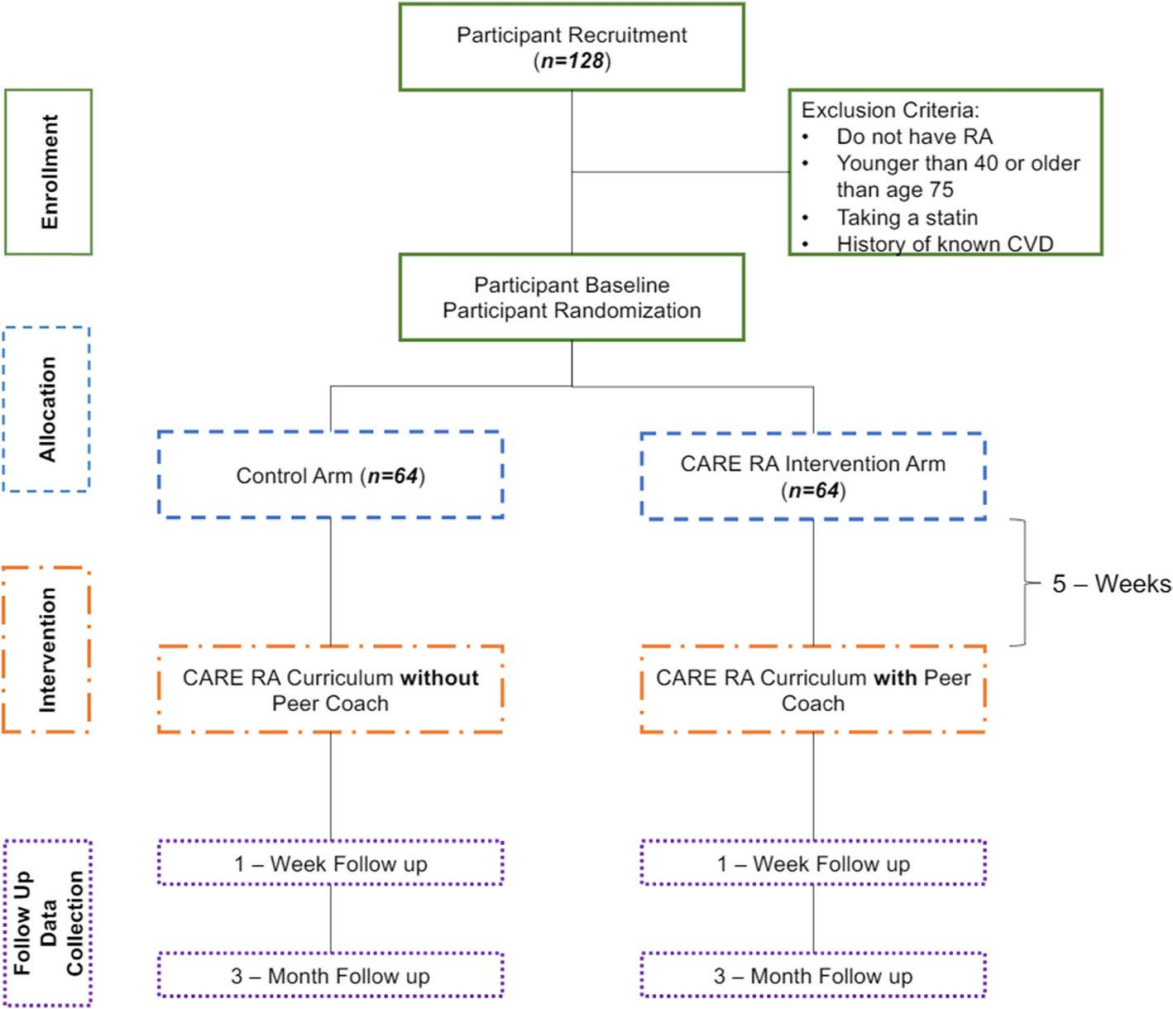
CONSORT diagram

This is an open-label, two-arm, randomized pilot trial that will test the feasibility of the peer coach intervention in anticipation of a larger trial that will facilitate the implementation of evidence-based cholesterol treatment guidelines among people with RA. Participants will be randomized 1:1 to either the peer coach intervention group or the control group (see CONSORT diagram in Fig. 1 and Additional file 2 for completed CONSORT checklist).

#### CARE-RA educational materials: the Patient Activated Learning System (PALS)

The educational materials for this study are available on the PALS website (www.palsforhealth.com) and are listed in Table 2. This novel education platform was developed at the Weill Cornell Medicine (WCM) Division of General Internal Medicine and the University of Alabama at Birmingham (UAB). It is based on Adult Learning Theory and Bandura’s SCT [42, 44]. The PALS is a publicly available educational and empowerment resource designed to provide engaging, easily understood, and well-researched facts for people who want to know more about health, medicines, and diseases [45]. The content in the PALS is evidence-based and peer-reviewed. This content is translated into patient-facing text in plain language, aiming for a seventh grade reading level. Some content is accompanied by visuals or short videos and a “sticky soundbite” to reinforce the single learning objective for each module, known in the PALS parlance as a renewable knowledge object (RKO). Each RKO includes an assessment question about the information that the reader has just reviewed.

**Table 2:** CARE RA peer coach intervention schedule and CARE RA education curriculum

Sessions	Objectives	PALS RKO/CARE RA education curriculum
**1: Introduction to the CARE RA Program**	1. Get to know each other 2. Introduce the CARE RA program 3. Introduce the client to the PALS	• None for Session 1
**2: RA and My Heart​**	1. Learn how RA affects your heart 2. Learn why a cholesterol test is needed to assess risk for heart attack and stroke	*Required:* • How can rheumatoid arthritis (RA) affect my body and my health? • What does atherosclerotic cardiovascular disease (ASCVD) risk mean? • How does rheumatoid arthritis (RA) affect my heart? • How can I lower my risk of heart disease if I have rheumatoid arthritis (RA)? • What is cholesterol? • Do I need a cholesterol test if I have rheumatoid arthritis (RA)? *Learn more:* • What is rheumatoid arthritis (RA)? • How is rheumatoid arthritis (RA) treated? What medicines are used to treat rheumatoid arthritis (RA)?
**3: The Different Doctors Caring for People with RA ​**	1. Learn what the difference between a rheumatologist and primary care provider is 2. Learn how important it is to have a primary care provider in addition to a rheumatologist if you have RA ​	*Required:* • What is a rheumatologist? • What is a primary care provider? • Do I need to see a primary care provider if I already see a rheumatologist for my rheumatoid arthritis (RA)? • What can a primary care provider do for me? • Which doctor should check my cholesterol levels if I have rheumatoid arthritis (RA)? *Learn more:* • What is a specialist or specialty doctor? What is the difference between a primary care provider and a specialist?
**4: Requesting a Cholesterol Test​**	1. Describe the importance of having a cholesterol test to learn what your risk is for heart attacks and strokes 2. Learn how to communicate with your doctor about getting your cholesterol checked 3. Provide an overview of the medications used to lower your risk for heart attacks and strokes	*Required:* • How can I talk with my doctor if they are in a rush? • What medications are used to treat high cholesterol? • Do statins lower my chances of blood pressure related problems like heart disease and stroke? • When do I have to start taking medicine to treat high cholesterol? • Can rheumatoid arthritis (RA) medications lower my risk for heart attacks and stroke? • Do I need to prepare for a cholesterol test? *Learn more:* • Can I take my rheumatoid arthritis (RA) medication with statins? What are some common side effects of statins?
**5: How Did it Go?​**	1. Discuss how your visit with the rheumatologist went and if you were able to request a cholesterol test 2. Learn about how exercise and diet can help people with RA lower their cholesterol 3. Establish a plan for following up with your primary care provider	*Required:* • What foods can help lower my cholesterol? • What is a healthy diet or eating pattern? • Can exercise help my rheumatoid arthritis (RA)? • How does exercise lower my risk for heart attacks and stroke if I have rheumatoid arthritis (RA)? • How can I exercise when I am in pain? • What should I expect when I receive the results of a cholesterol test? *Learn more:* • Can exercise make my rheumatoid arthritis (RA) symptoms worse? • What foods can help improve my rheumatoid arthritis (RA)? What are the benefits of exercise?

*CARE RA* Cardiovascular Risk Assessment for Patients with Rheumatoid Arthritis, *PALS* Patient Activated Learning System, *RKO* renewable knowledge object

#### Intervention: PALS with peer coach guidance

**Fig. 2:**
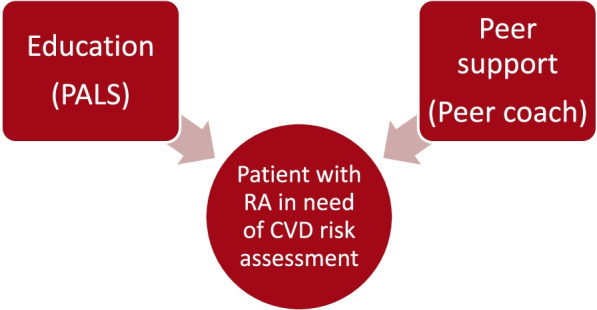
CARE RA components

The peer coach intervention consists of two main components: education and patient activation (Fig. 2). The education component is designed to develop an individual’s understanding of the relationship between RA and risk of CVD and will be available to all participants through the PALS. Those in the peer coach arm will be assigned a peer coach who will facilitate patient activation by developing competence in their participant on requesting a CVD risk assessment from their healthcare provider as well as willingness to participate in treatment decisions (e.g., initiating a statin). Participants will receive a digital copy of the CARE RA activity book and the intervention schedule. Peer coaches will have weekly 45-min calls with their participant to discuss the content of CARE RA for 5 weeks out of which four will be prior to the visit with the rheumatologist and one afterward. The session topics and how they map to SCT are shown in Table 1.

#### Power calculations and sample size

The trial is designed to have at least 80% power to detect an intervention-control difference of 20% for a two-sided test at a significance level of 0.05 for the primary outcome (CVD risk assessment or initiating a statin, if indicated). We assumed attrition would be 30%. These design parameters resulted in 64 participants in each group for a total of 128 participants. The modest sample size and relatively large intervention-control difference reflect the pilot nature of the trial.

#### Participant recruitment

We have several recruitment strategies. The first is to recruit participants from ArthritisPower, a Patient-Centered Outcomes Research Institute (PCORI)-funded Patient-Powered Research Network (PPRN). ArthritisPower is a collaboration between the nonprofit Global Healthy Living Foundation (GHLF), the parent organization of the CreakyJoints arthritis patient community, and researchers at the University of Alabama at Birmingham. Its registry includes over 30,000 adults who are interested in participating in research studies and who have RA, psoriatic arthritis, or other rheumatic, skin, and musculoskeletal conditions. ArthritisPower is overseen by Advarra IRB Protocol #201607783 [46].

The second is to invite patients from rheumatology clinics from Bendcare, a practice-based research network (PBRN) of 350 full-time community rheumatologists in 29 US states. At Bendcare, we will query the electronic health record (EHR) to select all patients with RA, age 40–75, not on a statin, with available email address, non-missing phone number, US residence, and no evidence of a cholesterol test in the past 2 years. Based on this list of pre-screened individuals, the staff from each practitioner’s office will send these individuals an invitation on their behalf to contact the research team of CARE RA. Once the individual contacts the research team, an unblinded research assistant will confirm eligibility and describe the study, and eligible individuals who remain interested will provide verbal informed consent. This informed consent will be voice recorded and saved on a HIPAA-compliant secure server of the WCM Division of General Internal Medicine.

#### Participant enrollment and randomization

After providing informed consent, participants will provide baseline data and then proceed to randomization. We will use REDCap to randomize participants 1:1 to either the control arm or the peer coach intervention arm. If randomized to the peer coach intervention arm, the participant will be matched with a peer coach, based on availability/schedules of both the participant and the peer coach. If randomized to the control arm, the participant will receive a brochure that includes the CARE RA curriculum from the PALS, and a tutorial on how to access the PALS. That is, control participants will receive all the information that is given to those in the peer coach intervention arm—the only difference is that those in the control arm will not have access to a peer coach.

#### Peer coach recruitment

Peer coaches will be recruited from several populations. Global Healthy Living Foundation research staff will identify candidates from the ArthritisPower PPRN who they believe would be appropriate peer coaches based on their leadership and experience within the online ArthritisPower community. Rheumatologists involved in the study will also refer patients from their rheumatology practice who may be suitable candidates to become peer coaches. Finally, it is anticipated that some participants who originally enter the study as clients will be recruited and trained to be peer coaches.

After an individual is referred, a research team member will determine whether the candidate meets inclusion criteria. All peer coaches must satisfy inclusion conditions for study participants. In addition, peer coaches must themselves have had, or be willing to have, a CVD risk assessment, must express interest in helping others with RA, and must be willing to discuss aspects of RA and CVD risk. Those who are taking a statin or have had a cholesterol test within the last 2 years will be considered to have met the risk assessment requirement. All other potential peer coaches are asked to have a cholesterol test and CVD risk assessment during peer coach training.

After screening, the research team will interview each peer coach candidate to assess their communication skills, volunteer experience, or previous experience in education or teaching. These experiences are important for identifying those with adequate communication proficiency for effective peer coaching. Experiences in education or volunteering are preferred but not required. The research team will review each candidate, and reasons for rejecting the candidate will be documented. An individual who is available and is considered a good candidate to be a peer coach will be scheduled for an upcoming training.

#### Peer coach training

Peer coach training is modeled on a previously published approach [47]. Two groups of 4 peer coaches will be scheduled for online training meetings for a total of 8 peer coaches. There will be a total of seven group web conference training sessions over 3 months, for 4 h a week. At the completion of the training, each peer coach will receive $500. At the first session, prospective peer coaches will be given an overview of the peer coach training schedule and CARE RA curriculum and intervention (Table 2). Subsequent sessions review each chapter of the Peer Coach manual to teach the activities that the peer coach will need to complete throughout the intervention and the checklist that they need to complete for each session (Additional file 1). Frequently asked questions (FAQs) are included to help peer coaches manage various situations that may arise with their client. The full FAQ are provided in Additional file 3. Before each training session, peer coaches-in-training are asked to review learning materials on PALS, listen to recordings of mock sessions between a peer coach and a client, review the CARE RA Activity Book, and review the relevant chapter in the Peer Coach Manual. During training meetings, peer coaches will receive a brief didactic education on Motivational Interviewing (MoI) skills, listen to mock sessions, practice delivering the session in turn to their partner, and discuss each other’s performance with everyone on the call to improve their skills [48].

Group web conferences will be supplemented by two MoI training meetings on videoconference with the research team and two coaches. During these smaller MoI meetings, peer coaches will practice MoI skills with reinforcement by the research team. Each MoI training meeting will include the following activities: (1) practicing MoI skills using role-playing scripts, alternating roles between coach and client under the supervision of the research team; (2) reinforcing skills with live feedback and encouragement, allowing peer coaches to critique each other’s skills and propose ways for improvement on techniques like “rolling with resistance,” “action planning,” and the use of the MoI-style open-ended questions, affirmations, reflective listening, and summaries.

Group trainings will be supplemented by paired practice. Each peer coach-in-training will be paired with another peer coach-in-training. Before the group trainings, the peer coaches-in-training will be asked to listen to a mock session on audio recordings. They will then each practice delivering the intervention in turn to their partner. Each coach-in-training will be encouraged to critique their partner’s performance based on their proficiency delivering the content and the use of MoI skills learned during training. If coaches do not feel confident that they have mastered a session, they will be encouraged to continue practicing with their partner. Once a coach-in-training feels confident, they will schedule a separate one-on-one certification test with the research team. At this test, the coach-in-training will deliver the intervention session to a member of the research team over the phone, who will play the role of the client, and a checklist will be used to assess proficiency (Additional file 4). Peer coaches will be approved as peer coaches by members of the research team once they receive scores of 90% or more in all sessions. If needed, additional training can be scheduled until each coach reaches a 90% score in their evaluation form. Only those who pass all the certifications for each intervention session will be allowed to work with clients as peer coaches in the study. Each peer coach will be receiving $20 per call that they complete with their client and can receive up to $100 per client that they guide through completing all the sessions of the CARE RA intervention. This payment is not contingent on the client’s requesting a cholesterol test or receiving a CVD risk assessment from their doctor.

#### Peer coach retention

Peer coach retention is a challenge for many peer coaching programs but is key to building a sustainable intervention. The strategies that CARE RA will use to retain coaches include ensuring timely payment for work and providing ongoing support, as well as continuing education/retraining with opportunities for practicing skills. All peer coach calls with clients will be recorded and monitored to evaluate the fidelity of the intervention. The ongoing support will include weekly group conference calls to assess fidelity to the CARE RA curriculum and monitor progress of the intervention. The weekly group conference calls will provide group problem-solving for challenges encountered, provide emotional support, and build group identity and pride. Each peer coach will determine their workload, increasing or decreasing according to life demands, which can enable them to continue to be part of the study in the face of changing personal circumstances. As time commitment can be an issue, the research team will aim to keep mandatory conference calls and individual calls on time, as short as possible, and reschedule only, if necessary, while aiding as needed in scheduling calls with participants.

### Study outcomes

#### Feasibility outcomes

We will determine the feasibility of the study by determining that at least 90% of the participants in the intervention arm complete all the sessions with the peer coach and that less or equal than 5% of the data that we intend to collect is missing. To enroll 128 participants, we expect to screen 500–600 individuals with RA. We will monitor recruitment success including screen failure rates to inform the design of the planned larger study to follow this one. Demographics of eligible individuals who chose not to participate will be compared to those who did choose to participate to estimate the magnitude of selection bias. Other feasibility outcomes include duration of the phone calls between peer coaches and clients, sustainability of peer coaches’ network, and retention of peer coaches. If this pilot study provides evidence for the feasibility of peer coach intervention in this population, we plan to launch a larger trial that focuses on getting statins initiated in patients with RA who also received an assessment for hypertension, diabetes, and hyperlipidemia and who met the criteria for statin initiation according to ACC/AHA. The future study will be powered only for the outcome of statin initiation. The strategies for the expansion will be determined by the results of this pilot study.

#### Clinical outcomes

The primary clinical outcome will be a participant having had a CVD risk assessment or initiating a statin, if indicated (Table 3). All the data collection and outcomes will be stored at Weill Cornell Medicine, Division of General Internal Medicine. Participants will complete the data collection survey 1 week after completing the intervention (e.g., aka, a week after their visit with their rheumatologist) and again 3 months after the visit with the rheumatologist. If participants have not completed the data collection surveys, a blinded member of the research team will follow up with a phone call to complete the forms over the phone and minimize missing data. We have designed the 3-month follow-up call to allow sufficient time for the participant to have the test and risk assessment and/or initiate medication following their visit with the rheumatologist. At each call, the participant will be asked whether they have received a risk assessment and whether they have initiated a statin. If we fail to get the information from the participant, the information will be retrieved, if possible, from the participant’s EHR. We will compare the proportion of participants in the intervention vs. control arm who report having had a risk assessment or initiated a statin.

**Table 3:** Data collection timelines and respective outcomes

	Baseline	1-week follow-up	3-month follow-up
Demographic (collected via EHR and patient survey)	X		
Medications and comorbidities (collected via EHR and patient survey)	X		
**Primary outcomes—patient-reported and/or electronic health record**			
CVD risk assessment (discuss with doctor the results of cholesterol test or having a cholesterol test, if not done at enrollment)	-	X	X
Initiating lipid-lowering therapy, if indicated	-	X	X
**Secondary outcomes—patient-reported**			
RAPID-3	X	X	X
Social Support Survey (SSS)	X	X	X
General Self-Efficacy Scale (GSE)	X	X	X
Patient Activation Measure (PAM)	X	X	X
Patient Health Questionnaire-8 (PHQ-8)	X	X	X
Medication Understanding and Use Self-Efficacy Scale (MUSE)	X	X	X

*CVD* cardiovascular disease, *EHR* electronic health record, *RAPID-3* Routine Assessment of Patient Index Data; 1-week and 3-week follow-up are reference to the appointment with the rheumatologist

Secondary outcomes will include patient activation and RA medication adherence. These outcomes will be measured, both at the 1-week follow-up and the 3-month follow-up, by changes from baseline to the end of the study in Routine Assessment of Patient Index Data (RAPID3), Social Support Survey Score, Patient Activation Measure (PAM), Patient Health Questionnaire - 8, General Self-Efficacy (GSE), and Medication Understanding and Use Self-Efficacy Scale (MUSE) [49–56]. We will compare these changes in the two trial arms.

### Implementation and program evaluation

We will use the RE-AIM Framework for the implementation and evaluation of the intervention. The RE-AIM framework provides a list of questions that helps researchers assess external validity and improves the likelihood of obtaining results that will have public health or population impact (Additional file 1). In addition, there are several issues of particular interest for the evaluation of this pilot study: (1) Geographic distribution: we plan to engage participants from the entire nation, rather than from only one site or one region of the country, as well as both urban and rural areas. (2) By using the PALS platform, we expect to be able to reach people who might not be able or willing to read long articles or pamphlets. (3) By recording calls between coaches and participants, we will be making every effort to guarantee that the intervention is delivered as it was intended to be delivered. It will be possible to determine whether the content delivered by the peer coaches is consistent with the content provided by the CARE RA curriculum hosted on the PALS website. Additional file 1 has details on the implementation and evaluation procedures of the intervention.

### Data analysis

To analyze the data, descriptive analyses (means, medians, standard deviations, proportions, and frequency distributions) and correlation analyses (Spearman/Pearson rank correlations) will be conducted to assess and describe the cohort. Baseline comparability between study arms and participants vs. non-participants will be assessed with parametric and nonparametric analyses including two-sample *t*-tests, Wilcoxon rank sum, and chi-square tests of proportions. Every effort will be made to keep participants in the study for the entire follow-up. Analysis will be based on intention-to-treat, regardless of whether the participant completed the intervention program. Two-sided *t*-tests will be used to calculate differences between the characteristics of the two arms and *p* values ≤ 0.05 will be considered statistically significant. We will determine differences in socio-demographics, physical function, and depressive symptoms. We will use last observation carry forward for missing data after the 1-week follow-up. Characteristics that are unbalanced by trial arm will be entered into a regression model examining the differences in the outcomes of the study by trial arm.

## Discussion

One goal of this study is to explore the effectiveness of a two-pronged peer coach intervention. The CARE RA intervention includes both an educational component, designed to improve participants’ understanding of the disease, and a training component, designed to improve participants’ ability to play an active role in their own medical care. The study is tightly focused on a single issue: CVD risk. But, compared to the general population, there is an increased prevalence among people with RA, not only of CVD, but also a number of other comorbidities, including infections, osteoporotic fracture, and lymphoma [57–60]. Many rheumatologists treating people with RA focus entirely on managing the disease and many primary care providers (PCPs) are unaware of the increased risk of comorbidities [28, 61]. These are barriers to the screening and treatment of comorbidities in general in this population. And it may well be that the best way to address these barriers is to provide tools for people with RA to play a more active role in managing their health care. Should the peer coach strategy prove efficacious on the restricted issue that this study tests, it will provide a promising strategy for addressing other problems in the health care of people with RA.

Another goal of this study is to contribute to empowering people with RA to play central roles as investigators in RA health care. One of the notable features of this study is the participation of people with RA as co-investigators in the design and development of the intervention rather than merely as research subjects. In recent years, it has become increasingly obvious to many researchers that it is important for stakeholders to be involved in carrying out medical research. This can be seen in the PCORI emphasis on stakeholder engagement—in particular, the Engagement Program Awards, which are designed to “encourage better integration of patients and other stakeholders into the research process” and in several recent articles about including stakeholders as co-authors in medical research [62–64]. The crucial role played by health care professionals in this research should not blind us to the possibility that there may be an equally crucial role for stakeholders to play, not just as research subjects, and not just as consultants who can inform investigators about the lived experience of a disease, but also as investigators. Unless stakeholders are true partners in the design and implementation of studies, there is abundant room for health care professionals to mistake the significance of the input of a stakeholder-consultant. If medical practice is to be changed in such a way as to enable stakeholders to play more of a role in their own medical care, the strategies for achieving this must answer to the actual needs and priorities of these stakeholders. We anticipate that the results of studies that involve stakeholders as investigators will do a better job of capturing priorities of stakeholders and, consequently, having more meaningful relevance for clinical practices than research carried out without participation of stakeholders.

### Innovations

The innovations of this study include (1) the heavy emphasis on patient-centeredness and a multi-stakeholder participatory model for the design and development of all materials; (2) the partnership with an online community and research registry, ArthritisPower, that comprises patients with rheumatic diseases who are interested in partnering with researchers, rheumatology providers, and other healthcare stakeholders to contribute to the advancement of clinical care and patient-centered research; (3) the use of peer coaches in rheumatic disease; (4) the use of patient activation and self-efficacy as the processes to develop and facilitate patient medical decision making; (5) the use of a novel platform for patient education and knowledge transfer, the PALS; and (6) the “direct-to-patient” approach of recruitment for a study like this as we are reaching out directly to patients [65].

This study allows us to learn new information on the perspective of patients with RA regarding CVD risk. The intervention model can be expanded in the future to include other CVD risk factors such as high blood pressure. The intervention will be designed to be patient-friendly because we are developing it with the direct input of people with RA and both academic and community rheumatologists.

### Strengths and limitations

The strengths of this study include the evidence-based nature of the CARE RA curriculum, which is informed by evidence-based guidelines endorsed by two national medical organizations, ACC and AHA. Another strength is its mapping to a theoretical framework (Social Cognitive Theory) and the use of an implementation framework (RE-AIM) to guide evaluation. An additional strength is the use of ArthritisPower, a well-established online community with a $2.6 million infrastructure investment PCORI to encourage and facilitate the conduct of exactly this type of research. Our collaboration with ArthritisPower and Bendcare in recruiting participants will allow for sampling throughout the nation and minimize the effect of geographic variations. Finally, another strength, as mentioned above, is that our study benefits from the extensive role played by stakeholders among our investigators.

Limitations include the small sample size, although this is to be expected in a pilot study. Another limitation is the population from which the first group of peer coaches is drawn. If the peer coaching strategy is to be used widely among people with RA, the peer coaches should be drawn from the general population of people with RA. However, it is anticipated that the initial group of peer coaches, which is comprised of activists, educators, and scientists, are more highly educated and more highly motivated than the general population from which peer coaches will ultimately be drawn. Thus, there may be barriers to training future peer coaches that will not be picked up in the pilot study. The population from which participants will be drawn constitutes another limitation to the study. Many of these participants will be drawn from the ArthritisPower registry, which is a patient-powered research network, and it may be that participants drawn from this registry are already inclined to be more proactive than most people with RA in obtaining a CVD risk assessment. If participants in both arms of the study are inclined to be proactive in obtaining a CVD risk assessment, the effect of the peer coach intervention may be underestimated. To address this, we will also engage community rheumatology practices to make sure that we have good representation of people with RA that might be less engaged in their medical care who are also included in the study.

## Conclusion

This study will develop a foundation to conduct a large pragmatic trial of this intervention (and future studies) in collaboration with ArthritisPower, academic institutions, and community practices of rheumatologists. This foundation will also be helpful in the development and implementation of future interventions that will incorporate other modifiable CVD risk factors, such as smoking cessation, hypertension, and weight loss and other comorbidities. It may help in the development of future interventions targeted at patients with other rheumatic diseases and other vexing challenges in rheumatology, such as medication/treatment adherence. Finally, we hope that it will be helpful generally, in addressing health care challenges for people with chronic diseases.

## Supplementary Information

**Additional file 1.** Detailed Measures Table.

**Additional file 2.** CONSORT 2010 checklist.

**Additional file 3.** CARE RA Peer Coach Frequently Asked Questions (FAQs).

**Additional file 4.** Checklist to assess proficiency.

## Data Availability

Data for the study design can be found in the manuscript figures, tables, and supplementary materials. For further information of study design, please email the corresponding author, Iris Navarro-Millán, MD MSPH at yin9003@med.cornell.edu.

## Abbreviations

ACC: American College of Cardiology
AHA: American Heart Association
CARE RA: Cardiovascular Risk Assessment for Rheumatoid Arthritis
CVD: Cardiovascular disease
DM: Diabetes mellitus
EHR: Electronic health records
FAQ: Frequently asked questions
GHLF: Global Healthy Living Foundation
GSE: General Self-Efficacy
HIPAA: Health Insurance Portability and Accountability Act of 1996
HIV: Human immunodeficiency virus
MoI: Motivational Interviewing
MUSE: Medication Understanding and Use Self-Efficacy Scale
PALS: Patient Activated Learning System
PAM: Patient Activation Measure
PBRN: Practice-Based Research Network
PCORI: Patient-Centered Outcomes Research Institute
PCP: Primary care provider
PPRN: Patient-Powered Research Network
RA: Rheumatoid arthritis
RAPID3: Routine Assessment of Patient Index Data
RE-AIM: Reach, Effectiveness, Adoption, Implementation, Maintenance
RKO: Renewable knowledge object
SCT: Social Cognitive Theory
UAB: University of Alabama, Birmingham
WCM: Weill Cornell Medicine
