# Supplementary material for: Protocol for the pilot randomized trial of the CArdiovascular Risk assEssment for Rheumatoid Arthritis (CARE RA) intervention: a peer coach behavioral intervention
Source: Pilot Feasibility Stud. 2022 Apr 15;8:84. doi: 10.1186/s40814-022-01041-z (PMC9011938; doi:10.1186/s40814-022-01041-z)
Supplement: Supplementary file 1 — Additional file 1. Detailed Measures Table. [file 40814_2022_1041_MOESM1_ESM.docx]

**Additional File 1:** Detailed Measures Table.

| **Name/Construct** | **Type (i.e., primary, secondary, other)** | **Time Frame** | **Description / Instrument** |
| --- | --- | --- | --- |
| CVD risk assessment | Primary/Effectiveness | 1-week follow up and 3-month follow up | Discuss with doctor the results of cholesterol test or having a cholesterol test, if not done at enrollment in the study.  Discussing cholesterol test results with their doctor will be self-reported. Having a cholesterol test done will be self-reported and/or obtained from electronic health record. |
| Initiating lipid-lowering therapy, if indicated | Primary/Effectiveness | 1-week follow up and 3-month follow up | This will be self-reported and/or obtained from electronic health record when possible. |
| RAPID - 3 | Secondary | Baseline, 1-week follow up and 3-month follow up | RAPID – 3 is a pooled index of the 3 patient-reported American College of Rheumatology rheumatoid arthritis (RA) Core Data Set measures: function, pain, and patient global estimate of status. Each of the 3 individual measures is scored 0 to 10, for a total of 30.^1,2^ |
| Social Support Survey | Secondary | Baseline, 1-week follow up and 3-month follow up | This survey was designed to be comprehensive in terms of recent thinking about the various dimensions of social support. Multitrait scaling analyses supported the dimensionality of four functional support scales (emotional/informational, tangible, affectionate, and positive social interaction) and the construction of an overall functional social support index. They are reliable (all Alphas greater than 0.91).^3^ |
| General Self-Efficacy (GSE) | Secondary | Baseline, 1-week follow up and 3-month follow up | General self-efficacy (GSE) is a reliable and valid instrument to assess this disposition. This scale is a self-report measure of self-efficacy of 10 items. The total score ranges between 10 and 40, with a higher score indicating more self-efficacy. Its reliability = Cronbach’s alphas between 0.76 and 0.90. GSE correlated to emotion, optimism, work satisfaction. Negative coefficients were found for depression, stress, health complaints, burnout, and anxiety.^4^ |
| Patient Activation Measure (PAM) | Secondary | Baseline, 1-week follow up and 3-month follow up | The Patient Activation Measure is a valid, highly reliable, unidimensional, probabilistic scale that reflects a developmental model of activation. Activation appears to involve four stages: (1) believing the patient role is important, (2) having the confidence and knowledge necessary to take action, (3) actually taking action to maintain and improve one's health, and (4) staying the course even under stress.^5^ |
| Patient Health Questionnaire – 8 (PHQ- 8) | Secondary | Baseline, 1-week follow up and 3-month follow up | The PHQ-8 is a useful depression measure for population-based studies, and either its diagnostic algorithm or a cut point > or = 10 can be used for defining current depression.^6^ |
| Medication Understanding and Use Self-Efficacy Scale (MUSE) | Secondary | Baseline, 1-week follow up and 3-month follow up | The MUSE is a valid and reliable tool measuring self-efficacy of understanding and using prescription medication (RA meds and statins if prescribed). This scale differs from existing medication-specific self-efficacy scales as it addresses both learning about one's medications and adherence to the prescribed regimen. The MUSE can be utilized among participants with varying literacy levels.^7^ |
| Demographics | Other | Baseline | Age, gender, race/ethnicity, education, insurance, income. |
| Rheumatoid arthritis | Other | Baseline | RA disease duration, patient global assessment of their rheumatoid arthritis, rheumatoid arthritis medications. |
| Social history | Other | Baseline | Smoking, vaping, alcohol consumption history. |
| Implementation climate | Feasibility | 3-week follow-up data collection | We will determine the number of participants that completed the study in the control arm and CARE RA arm and compare with how many initiate the study. The intervention will be considered feasible if more than 80% of enrolled participants complete the intervention and data collection points.  Retention of peer coaches in the study of 80% or more will be another metric that we will use for feasibility. |
| Implementation and Evaluation climate | Reach | Baseline | We will collect demographic and social data on all the participants that join the intervention. We will store the demographic and social information of individuals that drop out from the study. This information will provide us with an understanding of how the intervention is reaching a diverse group of people with rheumatoid arthritis. |
| Implementation and Evaluation climate | Adoption | After 3-week follow-up data collection (final encounter of the study) or after dropping out from the study | We will collect demographic and social data among all participants. We will also be inviting both participants that completed the study and those that drop out, to participate in semi-structured interviews regarding their experience with the study, the CARE RA curriculum, and working with a peer coach. This information will allow us to improve the adoption of the intervention in a larger clinical trial. |
| Implementation and Evaluation climate | Implementation/  Fidelity | Call of sessions 1-5 between peer coaches and clients. | All calls between peer coaches and clients will be recorded and reviewed by the research team to assess fidelity of the intervention.  Peer coaches must have discussed at least 80% of the items in the checklist of each of the sessions to make sure that the intervention is been delivered as intended. We will do additional training for peer coaches that are completing less than 80% of the corresponding session checklist. |
| Implementation and Evaluation climate | Maintenance | Weekly throughout the study (weekly calls with peer coaches) and after 3-week follow up data collection (final encounter of the study) | We will conduct semi-structured interviews to determine the best way to support participant and peer coach retention for the CARE RA program and how the processes in place were helpful or can be improved for maintenance. |

**References:**

1. Pincus T, Yazici Y, Bergman MJ. RAPID3, an index to assess and monitor patients with rheumatoid arthritis, without formal joint counts: similar results to DAS28 and CDAI in clinical trials and clinical care. *Rheum Dis Clin North Am.* 2009;35(4):773-778, viii.

2. Pincus T, Swearingen CJ, Bergman M, Yazici Y. RAPID3 (Routine Assessment of Patient Index Data 3), a rheumatoid arthritis index without formal joint counts for routine care: proposed severity categories compared to disease activity score and clinical disease activity index categories. *J Rheumatol.* 2008;35(11):2136-2147.

3. Sherbourne CD, Stewart AL. The MOS social support survey. *Soc Sci Med.* 1991;32(6):705-714.

4. Schwarzer R, Jerusalem, M. Generalized Self-Efficacy scale. In: J. Weinman SW, & M. Johnston, ed. *Measures in health psychology: A user’s portfolio. Causal and control beliefs*. Windsor, UK: NFER-NELSON.; 1995:35-37.

5. Hibbard JH, Stockard J, Mahoney ER, Tusler M. Development of the Patient Activation Measure (PAM): conceptualizing and measuring activation in patients and consumers. *Health services research.* 2004;39(4 Pt 1):1005-1026.

6. Kroenke K, Strine TW, Spitzer RL, Williams JB, Berry JT, Mokdad AH. The PHQ-8 as a measure of current depression in the general population. *Journal of affective disorders.* 2009;114(1-3):163-173.

7. Cameron KA, Ross EL, Clayman ML, et al. Measuring patients' self-efficacy in understanding and using prescription medication. *Patient Educ Couns.* 2010;80(3):372-376.
