# Supplementary material for: Protocol for the pilot randomized trial of the CArdiovascular Risk assEssment for Rheumatoid Arthritis (CARE RA) intervention: a peer coach behavioral intervention
Source: Pilot Feasibility Stud. 2022 Apr 15;8:84. doi: 10.1186/s40814-022-01041-z (PMC9011938; doi:10.1186/s40814-022-01041-z)
Supplement: Supplementary file 3 — Additional file 3. CARE RA Peer Coach Frequently Asked Questions (FAQs). [file 40814_2022_1041_MOESM3_ESM.docx]

**Additional File 3**: CARE RA Peer Coach Frequently Asked Questions (FAQs)

**What are the main outcomes the study team is looking at with the CARE RA program?**

The main outcomes of the study are the number of participants who receive CVD risk assessment at their upcoming rheumatologist appointment and the number of participants who have initiated a statin at the end of the 5-week intervention or the 3-month follow-up.

**Why is the CARE RA program considered a pilot study?**

Peer coaching interventions are gaining traction for chronic disease self-care. Peer coaches have been shown to improve medication adherence for human immunodeficiency virus (HIV), asthma, diabetes, and cancer screening. Peer coaches are lay individuals who themselves have the targeted chronic condition and who receive minimal training. They provide social and emotional support to the patient and could be used to help patients complete an educational program that includes decision support for CVD risk reduction strategies. However, peer coaches have not been used in RA, and have not been evaluated for CVD risk reduction in this population. For this reason, the CARE RA program is a pilot study to determine the effectiveness of peer coaching for people with RA who have a higher risk of heart attacks and strokes.

**Are there plans to expand the CARE RA program?**

There are plans to expand the CARE RA program, depending on the feasibility and effectiveness of the pilot program. We hope to expand to different demographics, including translating the program to Spanish and delivering to Spanish-speaking populations.

**How do I become a certified peer coach?**

To become certified as a peer coach you must complete an 8-week training program with our study team, which consists of 7 training sessions, 5 certification assessments, and 2 motivational interviewing sessions. You must also complete the onboarding material, Good Clinical Practice certification, and pre-and post-training surveys.

**How can I access the study materials?**

All study materials will be available to you through the Peer Coach Google Drive. The Google Drive will have the peer coach manual, activity book, peer coach notebook, video and audio files, and much more. You will receive a link through email to access the Drive. Keep the link in an accessible place or bookmark the link in your browser so you can easily access the Drive.

**Will the sessions with my client be recorded?**

Yes, every session with your client will be recorded and reviewed by a study team member to ensure that the content is being properly delivered and protocol is being followed.

**What is considered medical advice?**

Medical advice is considered any opinion directed towards another individual to diagnose and/or treat an existing or potential medical condition. For example, if your client states that their blood pressure is 189/112 and asks if that is high, you may not state that they have hypertension because that is your opinion on a diagnosis that you are not qualified to make. To be certain you are not providing medical advice to your client, direct any questions you aren’t sure about to the study team or direct your client to their health care providers.

**What do I do if I can’t reach my client?**

If you have attempted to reach your client more than 3 times for one session, or if your client is consistently tardy/late/unresponsive, please reach out to the study team. The study team will try to contact your client and remedy the situation.

**Can my client be kicked out of the program?**

Client retention is a vital part of the success of this program; however, your client may be kicked out of the program in extreme circumstances such as causing undue stress or harm to the peer coach.

**What happens if my client refuses to get a cholesterol test?**

If your client refuses to get a cholesterol test that is okay! Using your O.A.R.S. skills, you can help your client see the benefits of getting a cholesterol test but bringing these challenges to the weekly conferences can help you come up with additional strategies. If at the end of the 5-week intervention your client still decides to not get a cholesterol test, you will not face any consequences, and neither will the client. We will be following up with your client up to 3 months after they finish the program, and they may choose to receive a cholesterol test after they’ve had more time to think about it outside of the program. For this reason, it’s important to remain consistent in your messaging about receiving a cholesterol test through the end of the intervention.

**I’ve experienced an emotionally difficult conversation with my client, are there any resources for me that I can use?**

We value your efforts as a peer coach and appreciate your willingness to be vulnerable with your clients to help them with their journey through the program. Some conversations with your clients may trigger difficult memories or feelings. To move on from these difficult conversations, we welcome you to use the resources located in appendix E. We also encourage you to share your experience during our weekly conference meetings, communicate with other peer coaches through Slack for support, or you can request a meeting with the study team as well.

**My client is experiencing or has experienced some type of abuse (sexual abuse, elder abuse, etc.), what should I do?**

Given the complicated nature of mandatory reporting laws for different types of abuse, if your client expresses that they are currently experiencing or have experienced some type of abuse please alert the study team as soon as possible. In the meantime, provide your client with the support they need and direct them towards the mental health resources in appendix E of their activity book.
