# Supplementary material for: Protocol for the pilot randomized trial of the CArdiovascular Risk assEssment for Rheumatoid Arthritis (CARE RA) intervention: a peer coach behavioral intervention
Source: Pilot Feasibility Stud. 2022 Apr 15;8:84. doi: 10.1186/s40814-022-01041-z (PMC9011938; doi:10.1186/s40814-022-01041-z)
Supplement: Supplementary file 4 — Additional file 4. Checklist to assess proficiency. [file 40814_2022_1041_MOESM4_ESM.pdf]

PEER COACH: **DATE:** **ATTEMPT #:**

SESSION: 1 **CLIENT (PARTNER):**

EVALUATORS:

SESSION CONTENT (40%)

**Did the peer coach display the following competencies?**	**No**	**Needs Improvement**	**Yes**	**Comments**
Read over the Objectives and Agenda				
Verify the client has received the Activity Book, the PALS tutorial, and the program calendar				
Do "Get to know each other" activity with client				
Emphasize that you are not a medical professional and cannot provide medical advice				
Introduce the CARE RA Program				
Introduce the PALS				
Conduct PALS tutorial				
Review Rules and Responsibilities				
Sign Contract				
Discuss the PALS assignments for Session 2				
Discuss Learn More PALS optional assignments for Session 2				
Schedule Session 2 call				

OARS EVALUATION (20%)

**Did the peer coach display the following competencies?**	**No**	**Needs Improvement**	**Yes**	**Comments**
**OPEN-ENDED QUESTIONS**				
Did the coach ask open-ended questions?				
Did the coach keep communication moving forward?				
**AFFIRMATIONS**				
Did the coach provide affirmations towards the client?				
Were the coach's affirmations sincere?				
Did the coach acknowledge and validate the client's experiences and feelings?				
**REFLECTIVE LISTENING**				
Did the coach demonstrate reflective listening?				
Did the coach ask questions instead of assuming they understood the client?				
Did the coach show they had an interest in and respect for what the client had to say?				

Did they client demonstrate they accurately heard and understood their client?	◯	◯	◯	
**SUMMARIZE**	◯	◯	◯	
Did the coach summarize and reinforce the main points of the conversation?	◯	◯	◯	
Did the coach show they have been listening to the client?	◯	◯	◯	
Did the coach smoothly transition to the next topic?	◯	◯	◯	

GENERAL MANNER (40%)

	No	Needs Improvement	Yes	Comments
Did the coach arrive to their certification session on time?	◯	◯	◯	
Was the coach supportive to their client?	◯	◯	◯	
Was the coach kind to their client?	◯	◯	◯	
Did the coach connect well with their client?	◯	◯	◯	

What were the coach's strengths during this certification session?

What are some areas the coach can improve upon from this certification session?

What feedback does the coach have for the study team regarding session 1? This includes feedback on training, manual, activity book, and other study materials.

Did the coach pass this certification session? ☐

PEER COACH: **DATE:** **ATTEMPT #:**

SESSION: 2 **CLIENT (PARTNER):**

EVALUATORS:

SESSION CONTENT (40%)

**Did the peer coach display the following competencies?**	**No**	**Needs Improvement**	**Yes**
Read over the Objectives and Agenda	◯	◯	◯
Verify the client has reviewed the RKOs	◯	◯	◯
Review the PALS assignments	◯	◯	◯
Read through CARE RA Highlights	◯	◯	◯
Discuss plans for Session 3	◯	◯	◯
Discuss PALS assignments for Session 3	◯	◯	◯
Discuss Learn More PALS assignments for Session 3	◯	◯	◯
Schedule Session 3 call	◯	◯	◯

OARS EVALUATION (20%)

**Did the peer coach display the following competencies?**	**No**	**Needs Improvement**	**Yes**
**OPEN-ENDED QUESTIONS**	◯	◯	◯
Did the coach ask open-ended questions?	◯	◯	◯
Did the coach keep communication moving forward?	◯	◯	◯
**AFFIRMATIONS**	◯	◯	◯
Did the coach provide affirmations towards the client?	◯	◯	◯
Were the coach's affirmations sincere?	◯	◯	◯
Did the coach acknowledge and validate the client's experiences and feelings?	◯	◯	◯
**REFLECTIVE LISTENING**	◯	◯	◯
Did the coach demonstrate reflective listening?	◯	◯	◯
Did the coach ask questions instead of assuming they understood the client?	◯	◯	◯
Did the coach show they had an interest in and respect for what the client had to say?	◯	◯	◯
**SUMMARIZE**	◯	◯	◯
Did the coach summarize and reinforce the main points of the conversation?	◯	◯	◯
Did the coach smoothly transition to the next topic?	◯	◯	◯

GENERAL MANNER (40%)

	No	Needs Improvement	Yes
Did the coach arrive to their certification session on time?	○	○	○
Was the coach supportive to their client?	○	○	○
Was the coach kind to their client?	○	○	○
Did the coach connect well with their client?	○	○	○

What were the coach's strengths during this certification session?

What are some areas the coach can improve upon from this certification session?

What feedback does the coach have for the study team regarding session 1? This includes feedback on training, manual, activity book, and other study materials.

Did the coach pass this certification session? ☐

PEER COACH: **DATE:** **ATTEMPT #:**

SESSION: 3 **CLIENT (PARTNER):**

EVALUATORS:

SESSION CONTENT (40%)

**Did the peer coach display the following competencies?**	**No**	**Needs Improvement**	**Yes**
Introduction to Session 3	◯	◯	◯
Read over the Objectives and Agenda	◯	◯	◯
Verify RKOs are completed	◯	◯	◯
Review RKOs for Session 3	◯	◯	◯
Discuss CARE RA Highlights for Session 3	◯	◯	◯
Making a Plan: Part 1	◯	◯	◯
Assignments for Session 4	◯	◯	◯
Schedule Session 4 call	◯	◯	◯

OARS EVALUATION (20%)

**Did the peer coach display the following competencies?**	**No**	**Needs Improvement**	**Yes**
**OPEN-ENDED QUESTIONS**	◯	◯	◯
Did the coach ask open-ended questions?	◯	◯	◯
Did the coach keep communication moving forward?	◯	◯	◯
**AFFIRMATIONS**	◯	◯	◯
Did the coach provide affirmations towards the client?	◯	◯	◯
Were the coach's affirmations sincere?	◯	◯	◯
Did the coach acknowledge and validate the client's experiences and feelings?	◯	◯	◯
**REFLECTIVE LISTENING**	◯	◯	◯
Did the coach demonstrate reflective listening?	◯	◯	◯
Did the coach ask questions instead of assuming they understood the client?	◯	◯	◯
Did the coach show they had an interest in and respect for what the client had to say?	◯	◯	◯
**SUMMARIZE**	◯	◯	◯
Did the coach summarize and reinforce the main points of the conversation?	◯	◯	◯
Did the coach smoothly transition to the next topic?	◯	◯	◯

GENERAL MANNER (40%)

	No	Needs Improvement	Yes
Did the coach arrive to their certification session on time?	○	○	○
Was the coach supportive to their client?	○	○	○
Was the coach kind to their client?	○	○	○
Did the coach connect well with their client?	○	○	○

What were the coach's strengths during this certification session?

What are some areas the coach can improve upon from this certification session?

What feedback does the coach have for the study team regarding session 1? This includes feedback on training, manual, activity book, and other study materials.

Did the coach pass this certification session? ☐

PEER COACH: **DATE:** **ATTEMPT #:**

SESSION: 4 **CLIENT (PARTNER):**

EVALUATORS:

SESSION CONTENT (40%)

**Did the peer coach display the following competencies?**	**No**	**Needs Improvement**	**Yes**
Introduction to Session 4	☐	☐	☐
Review RKOs for Session 4	☐	☐	☐
Discuss CARE RA Highlights	☐	☐	☐
Making a Plan: Part 1 Discussion	☐	☐	☐
Making a Plan: Part 2	☐	☐	☐
Rehearse Scripts	☐	☐	☐
Assignments for Session 5	☐	☐	☐
Schedule Session 5 call	☐	☐	☐

OARS EVALUATION (20%)

**Did the peer coach display the following competencies?**	**No**	**Needs Improvement**	**Yes**
**OPEN-ENDED QUESTIONS**			
Did the coach ask open-ended questions?	☐	☐	☐
Did the coach keep communication moving forward?	☐	☐	☐
**AFFIRMATIONS**			
Did the coach provide affirmations towards the client?	☐	☐	☐
Were the coach's affirmations sincere?	☐	☐	☐
Did the coach acknowledge and validate the client's experiences and feelings?	☐	☐	☐
**REFLECTIVE LISTENING**			
Did the coach demonstrate reflective listening?	☐	☐	☐
Did the coach ask questions instead of assuming they understood the client?	☐	☐	☐
Did the coach show they had an interest in and respect for what the client had to say?	☐	☐	☐
**SUMMARIZE**			
Did the coach summarize and reinforce the main points of the conversation?	☐	☐	☐
Did the coach smoothly transition to the next topic?	☐	☐	☐

GENERAL MANNER (40%)

	No	Needs Improvement	Yes
Did the coach arrive to their certification session on time?	☐	☐	☐
Was the coach supportive to their client?	☐	☐	☐
Was the coach kind to their client?	☐	☐	☐
Did the coach connect well with their client?	☐	☐	☐

What were the coach's strengths during this certification session?

What are some areas the coach can improve upon from this certification session?

What feedback does the coach have for the study team regarding session 4? This includes feedback on training, manual, activity book, and other study materials.

Did the coach pass this certification session? ☐

PEER COACH: **DATE:** **ATTEMPT #:**

SESSION: 5 **CLIENT (PARTNER):**

EVALUATORS:

SESSION CONTENT (40%)

**Did the peer coach display the following competencies?**	**No**	**Needs Improvement**	**Yes**
Introduction to Session 5	☐	☐	☐
Verify RKOs are Completed	☐	☐	☐
Review RKOs for Session 5	☐	☐	☐
Discuss CARE RA Highlights for Session 5	☐	☐	☐
Discuss Making a Plan: Part 2	☐	☐	☐
Questions and Closure	☐	☐	☐

OARS EVALUATION (20%)

**Did the peer coach display the following competencies?**	**No**	**Needs Improvement**	**Yes**
**OPEN-ENDED QUESTIONS**			
Did the coach ask open-ended questions?	☐	☐	☐
Did the coach keep communication moving forward?	☐	☐	☐
**AFFIRMATIONS**			
Did the coach provide affirmations towards the client?	☐	☐	☐
Were the coach's affirmations sincere?	☐	☐	☐
Did the coach acknowledge and validate the client's experiences and feelings?	☐	☐	☐
**REFLECTIVE LISTENING**			
Did the coach demonstrate reflective listening?	☐	☐	☐
Did the coach ask questions instead of assuming they understood the client?	☐	☐	☐
Did the coach show they had an interest in and respect for what the client had to say?	☐	☐	☐
**SUMMARIZE**			
Did the coach summarize and reinforce the main points of the conversation?	☐	☐	☐
Did the coach smoothly transition to the next topic?	☐	☐	☐

GENERAL MANNER (40%)

	No	Needs Improvement	Yes
Did the coach arrive to their certification session on time?	☐	☐	☐
Was the coach supportive to their client?	☐	☐	☐
Was the coach kind to their client?	☐	☐	☐
Did the coach connect well with their client?	☐	☐	☐

What were the coach's strengths during this certification session?

What are some areas the coach can improve upon from this certification session?

What feedback does the coach have for the study team regarding session 5? This includes feedback on training, manual, activity book, and other study materials.

Did the coach pass this certification session? ☐

PEER COACH:

PARTNER (CLIENT):

DATE:

MI SESSION #:

OBSERVERS:

FEEDBACK

Open-Ended Questions

Affirmations

Reflective Listening

Summarize
